# Physiological Quality of Bean Seeds Cultivated with Rhizobia Reinoculation and *Azospirillum* Co-Inoculation at Different Growth Stages

**DOI:** 10.3390/microorganisms13040805

**Published:** 2025-04-01

**Authors:** Nathan Mickael de Bessa Cunha, Itamar Rosa Teixeira, Gisele Carneiro da Silva Teixeira, Ednaldo Cândido Rocha, Tamires Ester Peixoto Bravo, Andressa Laís Caldeira de Souza, Eulina Fernandes Damião, Alexandre Marcos Sbroggio Filho

**Affiliations:** 1 Institute of Agricultural Science, State University of Goiás, Anápolis 75132-903, GO, Brazil; nathan.cunha@aluno.ueg.br (N.M.d.B.C.); gisele.carneiro@ueg.br (G.C.d.S.T.); ednaldo.rocha@ueg.br (E.C.R.); tamires.bravo@aluno.ueg.br (T.E.P.B.); andressa_agro1996@outlook.com (A.L.C.d.S.); eulina@aluno.ueg.br (E.F.D.); 2 Department of Agronomy, Federal University of Goiás, Goiânia 74690-900, GO, Brazil; alexandremarcos@discente.ufg.br

**Keywords:** *Rhizobium tropici*, *Azospirillum brasilense*, seed vigor, germination, inoculation, co-inoculation, common bean

## Abstract

This study evaluates the impact of *Rhizobium tropici* reinoculation and *Azospirillum brasilense* co-inoculation at different growth stages on the physiological quality of common bean seeds. A randomized block design was used, assessing germination, vigor, electrical conductivity, seedling length, and dry mass. Treatments T7 (co-inoculation *R. tropici* + *A. brasilense* at R5) showed the highest germination rates, indicating enhanced seed viability. The accelerated aging test revealed that T7 exhibited greater resistance to stress, presenting greater seedling vigor, whereas T10 and T11 were more susceptible. The electrical conductivity results remained stable across treatments, suggesting that cell membrane integrity was not significantly compromised. Seedling length and dry mass did not present significant variations, reinforcing the idea that early germination and vigor are primary indicators of seed quality. Canonical discriminant analysis and MANOVA confirmed significant treatment differences, highlighting the influence of inoculation strategies on seed physiology. Overall, co-inoculation with *Rhizobium tropici* and *Azospirillum brasilense* (particularly in T7) demonstrated potential to improve seed quality at lower cost, offering sustainable alternatives for optimizing agricultural production.

## 1. Introduction

Agriculture faces constant challenges both in the field and in society to balance productivity and sustainability in food production. Among Brazil’s main crops, common bean (*Phaseolus vulgaris* L.) stands out as a key player in both national nutrition and the economy. As one of the most important legumes in the Fabaceae family, this grain is a vital source of income for small and medium-sized farmers and remains an essential food on the plates of most Brazilians.

In recent years, Brazil has established itself as one of the world’s largest bean producers. According to data from the National Supply Company [1], during the 2023/2024 harvest, bean cultivation covered an area of 2.8563 million hectares, resulting in a total production of 3.2493 million tons. The average productivity reached 1.138 kg per hectare, highlighting the significance of this crop for national agriculture.

The physiological quality of soybean [2] and sorghum [3] seeds is directly linked to the availability of nutrients for the mother plant. Furthermore, this factor influences the storage of essential compounds, such as proteins, carbohydrates, lipids, and minerals, ultimately affecting the productive potential of seeds [4]. Nutritional balance plays a fundamental role in the formation of the embryo and cotyledons [5].

According to [6], the application of an inoculant containing *Rhizobium tropici* in the planting furrow, followed by reinoculation at the V4 stage, resulted in the production of higher-quality bean seeds, with germination rates comparable to those obtained with nitrogen mineral fertilization (96% and 94%, respectively). In contrast, the isolated inoculation of *R. tropici* via seed was not sufficient to ensure seed lots of superior quality.

The co-inoculation technique has the potential to enhance both seed productivity and quality. According to [7], the co-inoculation of *Bradyrhizobium* and *Azospirillum* directly on soybean seeds promotes an increase in nodulation and shoot biomass development, which benefits grain yield components. Additionally, this practice significantly contributed to seed physiological quality, resulting in a 16% increase over three growing seasons compared to the control. In agreement with [8], inoculation with *Bacillus amyloliquefaciens* and mycorrhizae enhances soybean tolerance to water stress, improving biomass, yield, and seed quality even under drought conditions.

Recent studies have analyzed co-inoculation without considering different phenological stages, while previous research has primarily focused on inoculation at sowing, without exploring the impact of reinoculation timing on seed physiological quality. Furthermore, most studies have evaluated only vegetative and yield parameters, without investigating its effects on the next generation. This study advances knowledge by incorporating the temporal factor and directly analyzing the quality of the produced seeds.

Thus, ensuring adequate nutrition for mother plants throughout the entire cultivation cycle is essential, as it affects both nutrient accumulation in the aerial parts and their subsequent transfer and deposition in the seeds [9].

The search for improvements in seed quality and yield has motivated several studies [10]. In this context, practices such as rhizobial reinoculation, using bacteria capable of fixing atmospheric nitrogen, and co-inoculation with *Azospirillum*, a plant growth-promoting microorganism, have emerged as promising strategies to enhance bean productivity [11,12].

Reinoculation ensures a continuous supply of microorganisms, especially in soils where *rhizobium* persistence is low [13]. Studies indicate that single inoculation may be limited by competition with native microbiota and environmental factors [14]. Under field conditions, reinoculation is often necessary to maintain effective cell densities [15].

Rhizobial reinoculation consists of applying these bacteria to the soil at different stages of plant growth. This technique aims to maximize biological nitrogen fixation, an essential process for ensuring vigorous development and higher productivity in bean cultivation. Studies suggest that this practice can significantly enhance plant nodulation, resulting in healthier and more productive plants [16,17]

At the same time, co-inoculation with *Azospirillum* has gained increasing attention due to its multiple agronomic benefits. This bacterium promotes plant growth by stimulating phytohormone production, solubilizing phosphates, and improving nutrient absorption [18]. When combined with rhizobia, this interaction can amplify the positive effects on bean plants, fostering greater vigor and seed productivity [19].

Seed physiological quality is a crucial aspect of agricultural success, directly influencing germination, vigor, and seedling uniformity [20]. Practices involving beneficial microorganism inoculation have the potential to improve these attributes [21]. Furthermore, adopting techniques that optimize soil resource use and reduce fertilization costs presents a viable and low-cost alternative for seed production [22]. Evaluating the efficacy of rhizobia reinoculation and *Azospirillum* co-inoculation at different growth stages of common beans provides valuable insights for improving agricultural practices and achieving sustainable productivity [17].

When applying these biotechnological approaches, it is essential to consider the timing of inoculation, as the plant’s growth stage can influence the efficiency of microbial colonization and, consequently, the benefits provided to the plants [23]. Further investigation into nutrition and the production of high quality seeds is necessary, given that seed quality is closely related to the nutritional status of the mother plant and may, therefore, be influenced by the cultivation environment [24].

Adopting a strategy that integrates these practices could serve as a model for sustainable agriculture, contributing to the reduction in chemical fertilizer dependence and promoting soil health. This study aimed to evaluate the effects of *Rhizobium tropici* reinoculation and co-inoculation with *Azospirillum brasilense* at different phenological stages of common bean on seed physiological quality. Our hypothesis is that co-inoculation during reproductive stages may enhance biological nitrogen fixation and, consequently, promote seed germination and vigor of common bean.

## 2. Materials and Methods

### 2.1. Characterization of the Study Area

The experiment was conducted in two phases: (i) in the field and (ii) at the Plant Analysis Laboratory (LABAV), located at the Research and Graduate Studies Center (CPPG) of UEG/CCET. The field phase took place during the 2023/2024 “rainy season” growing cycle at the experimental area of the Goiás Agency for Technical Assistance, Rural Extension, and Agricultural Research—EMATER, in Anápolis, Goiás, located at coordinates 16°20′12.13″ S and 48°53′15.96″ W, at an average altitude of 1058 m [25].

**Figure 1:**
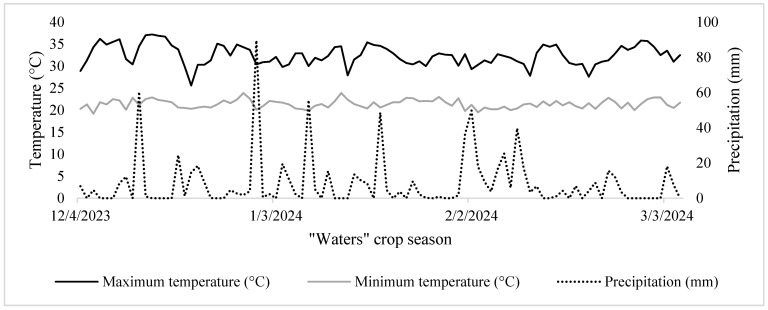
Climatic data on maximum and minimum temperatures and precipitation for the period from December 2023 to March 2024 in the municipality of Anápolis, Goiás, Brazil.

According to the Köppen climate classification, the local climate is tropical humid (Aw), with a dry season in the autumn and winter months and rainfall during the spring and summer months. The region has an average annual temperature of 22 °C and an average annual precipitation of 1677 mm [26]. Precipitation and daily average temperature data for the rainy-season cultivation period are presented in Figure 1.

The soil in the experimental area was classified as a dystroferric Red Latosol. Soil samples were collected from the 0–20 cm layer and sent to a laboratory for analysis, where chemical and physical characterizations were performed. The results are presented in Table 1.

### 2.2. Experimental Design and Treatments

The field experiment followed a randomized block design with four repetitions. The treatments tested in the field study are described in Table 2.

The inoculated treatments initially received liquid commercial [Biomax^®^ Premium liquid recommended for common bean] inoculants applied directly to the seeds, containing *Rhizobium tropici* cells (Strains SEMIA 4088 and SEMIA 4077) at a concentration of 2 × 10⁹ CFU/mL, using 150 mL per 50 kg of seeds. Co-inoculation with liquid commercial *Azospirillum brasilense* cells [Biomax^®^ Azum, recommended for various crops including bean] (strains AbV5) was carried out at a dose of 100 mL per 50 kg of seeds, following manufacturer recommendations for common bean crops. After the initial inoculation, these treatments were subjected to reinoculations using triple doses to enhance the interaction of biological products with soil particles [27,28].

Additionally, four treatments were tested: a control (without nitrogen fertilization), seeds inoculated with *R. tropici*, seeds co-inoculated with *A. brasilense*, and mineral nitrogen fertilization with 16 kg ha^−1^ of N at planting and 64 kg ha^−1^ as topdressing.

### 2.3. Field Experiment Implementation and Conduct

The experimental plots consisted of four rows, each 5 m long, spaced 0.50 m apart. The two central rows were used as the useful area, while the two outer rows served as borders. Based on soil analysis, liming was carried out by applying 2.0 tons per hectare of dolomitic limestone to correct high soil acidity (Table 1). Three months after liming, the soil was prepared using conventional tillage, with one plowing and two harrowings. Basal fertilization was applied to all plots using 400 kg ha^−1^ of 4-30-20 fertilizer in the planting furrow refers to a formulation containing 4% nitrogen (N), 30% phosphorus (P_2_O_5_), and 20% potassium (K_2_O), used to supply essential nutrients for the early development of common bean.

The planting density was 12 plants per meter. The cultivar used was BRS Estilo, which has carioca-type grains, upright growth habit (Type II), an average cycle of 90 days, and is suitable for mechanized harvesting. In addition, it is resistant to common mosaic and shows intermediate resistance to anthracnose and rust. It has a productive potential of 4 tons per hectare [29].

Reinoculations were carried out between stages V4 and R6, using a 5 L sprayer with a jet directed to the soil to deposit the bacteria in the root system. Topdressing nitrogen fertilization with urea (80 kg ha^−1^) was applied in mineral nitrogen fertilization treatments, split into two applications at 25 and 35 days after emergence (DAE) to minimize volatilization and leaching losses.

### 2.4. Laboratory Experiment Implementation and Conduct

After manual harvesting, the plants were sun-dried and manually threshed. Seeds were cleaned with sieves and stored in paper bags under dry conditions (approximately 20 °C and 40% relative humidity). The seed moisture content was determined at 105 ± 3 °C for 24 h using five replicates of 50 seeds, following the Rules for Seed Testing (RAS) [30]. Subsequently, the following evaluations were carried out with four repetitions each:

#### 2.4.1. Germination Test

Four subsamples of 50 seeds per treatment were placed in germitest paper rolls moistened with water equivalent to 2.5 times the paper’s mass. The rolls were maintained in a germinator at 25 ± 1 °C, with counts performed on the fifth and eighth days. Results were expressed as the percentage of normal seedlings, following RAS criteria [30].

#### 2.4.2. First Count

Conducted alongside the germination test, this involved recording the percentage of normal seedlings on the fifth day after test setup [30]. This count is considered a vigor test, reflecting the potential for rapid seedling emergence and development of normal seedlings [30].

#### 2.4.3. Accelerated Aging

A single layer of 300 seeds per treatment was evenly distributed on a screen placed inside a gerbox containing 40 mL of water and maintained in a BOD (*Biochemical Oxygen Demand*) chamber at 42 °C for 48 h, equivalent to the stressful field conditions (high temperature and humidity) encountered by the crop. After this period, the seeds underwent the germination test, as described earlier, and the normal seedlings were evaluated five days later.

#### 2.4.4. Electrical Conductivity

This test evaluated the electrical conductivity of the seed imbibition solution. Four replicates of 50 seeds, without visible damage, were soaked in plastic cups (200 mL capacity) containing 75 mL of distilled water for 24 h at 25 °C. Conductivity was measured using a TEC 4 MP digital conductivity meter, and the results were expressed in μS cm^−1^ g^−1^ of seed [31].

#### 2.4.5. Seedling Length

Seedling length was assessed using germitest paper rolls moistened according to germination test recommendations. Ten seeds per replicate were used. After five days, seedlings were measured with a graduated ruler in millimeters, considering both shoot and main root lengths. Results were expressed in centimeters (with one decimal place), and the mean for each replicate was calculated as the arithmetic average.

#### 2.4.6. Seedling Dry Mass

After the seedling length test, seedlings from each treatment were placed in kraft paper bags and dried in a forced-air oven at 65 °C for 48 h. Once dried, samples were cooled and weighed using a precision balance. Results were expressed as seedling dry mass (mg seedling^−1^).

### 2.5. Statistical Analysis

Data were subjected to multivariate analysis of variance (MANOVA) and Canonical Discriminant Analysis (CDA) to evaluate group separation based on seed physiological variables: first count (FC), germination (GER), accelerated aging (AA), electrical conductivity (EC), seedling length (SL), and seedling dry mass (SDM). Additionally, Pearson’s correlation was used to identify relationships between variables. All analyses were performed using R software (version 4.3.1) with a 5% significance level (*p* < 0.05).

## 3. Results

### 3.1. Mean Comparison

The first count (Table 3) varied across treatments, with T7 showing the highest mean (90.50%), followed by T10 (86.00%) and T9 and T12 (83.50%). Treatments such as T4 (28.50%) and T5 (57.50%) showed significant reductions in the first count, indicating potential negative impacts on their initial germination capacity. Overall, treatments involving co-inoculation T7 and inoculation T10 demonstrated superior performance, suggesting that co-inoculation and inoculation can enhance the physiological quality of seeds.

The general germination results followed a pattern similar to the first count (Table 3), with T10 and T7 standing out as the highest-performing treatments (95.50% and 94.00%, respectively). Treatments such as T4 (76.00%) and T5 (77.50%) showed relatively low germination values, possibly associated with lower seed vigor. The presence of *R. tropici* and *A. brasilense* at the inoculation doses, particularly in treatments T10 and T7, likely contributed to improved germination rates.

The accelerated aging test revealed significant differences among treatments. T7 achieved the highest value (39.00%), indicating greater resistance to aging stress. Conversely, T11 and T10 showed the lowest resistance (2.25% and 2.50%, respectively), which may reflect greater susceptibility to environmental stress. Treatments T2, T3, and T6 exhibited intermediate values, suggesting moderate performance under stress conditions.

It is worth noting that T10 (inoculation via seed) ensured efficient early colonization, favoring seed germination. However, these results were obtained in tests conducted under controlled conditions (e.g., germination and first count), and therefore less realistic with field conditions, where the crop is subject to varying climatic conditions. In this case, the accelerated aging test, since it is conducted under stressful conditions (high temperature and humidity), becomes more realistic with field conditions, and it was precisely in this test that the best seed vigor was again observed in T7, while T10 presented the worst result, a trend that was confirmed in the first count test conducted under controlled conditions together with the germination test.

Electrical conductivity did not show significant variation between treatments, with most values around 100 μS cm^−1^ g^−1^. Treatments T6 and T8 recorded the lowest EC values (83.75 μS cm^−1^ g^−1^ and 87.50 μS cm^−1^ g^−1^, respectively), which may indicate greater cellular membrane integrity and lower leaching of soluble compounds, suggesting higher seed quality in these treatments. On the other hand, T10 presented the highest average CE value among the treatments evaluated, despite not differing statistically from the others. These results partly confirm the data obtained in the accelerated aging test.

Seedling length did not differ significantly between treatments, ranging from 17.75 cm (T11) to 22.25 cm (T9). Although treatment T9 showed the highest value, followed by T2 and T12, these results do not indicate a consistent effect of inoculation on early seedling development. However, there was a tendency for treatment T10, followed by T11, to present lower average seedling lengths, confirming the results of the aging and electrical conductivity tests.

Seedling dry mass varied from 0.17 mg (T3) to 0.21 mg (T12). In general, there were no significant variations, with treatments displaying very similar averages. This indicates that dry mass was not strongly influenced by the treatments, suggesting that other factors, such as germination quality, may have played a more prominent role in determining seedling vigor.

### 3.2. Canonical Discriminant Analysis

**Figure 2:**
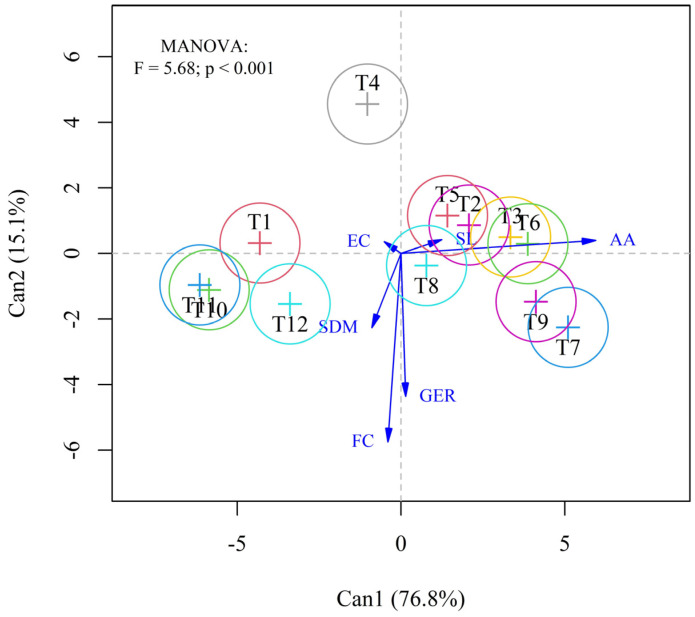
Canonical discriminant analysis of different inoculation and co-inoculation treatments with *Rhizobium tropici* and *Azospirillum brasilense* in common bean, based on seed physiological quality variables: first count (FC), germination (GER), accelerated aging (AA), electrical conductivity (EC), seedling length (SL), and seedling dry mass (SDM).

In canonical discriminant analysis in Figure 2, the first canonical variable (Can1), accounting for 76.8% of the explained variation, exhibited a strong association with the variables first count (FC) and germination (GER). In contrast, the second canonical variable (Can2), which explains 15.1% of the variation, was more strongly related to electrical conductivity (EC) and seedling dry mass (SDM). Together, these two canonical variables accumulated 91.9% of the total explained variation, indicating a robust model for treatment discrimination.

The evaluated treatments exhibited distinct behaviors. Reinoculation with *Rhizobium tropici* (T1, T2, T3, and T4) and co-inoculation with *Azospirillum brasilense* (T5, T6, T7, and T8), performed at the phenological stages V3, V4, R5, and R6, showed differentiated patterns in the distribution of treatments. Treatment T4 (reinoculation at stage R6) stood out for its strong association with accelerated aging, suggesting greater physiological seed deterioration when applied at later phenological stages. On the other hand, treatment T8 (co-inoculation at stage R6) exhibited lower correlations with the analyzed variables, positioning itself in an intermediate region of the graph.

Another highlight was mineral nitrogen fertilization (T12), which showed a strong relationship with EC and SDM, indicating superior performance in vigor and initial seedling quality. Treatments T1 (reinoculation at V3) and T10 (*Rhizobium tropici* inoculation via seed) clustered near T12, suggesting similar effects on initial vigor, while T2 and T6 exhibited more balanced correlations among GER, EC, and SDM.

The MANOVA results confirmed statistically significant differences among treatment groups (F = 5.68; *p* < 0.001), supporting the separation observed in the discriminant model. These findings highlight the impact of different inoculant application techniques, particularly concerning variables related to initial vigor and the physiological quality of seeds produced using these methods.

### 3.3. Pearson Correlation

**Figure 3:**
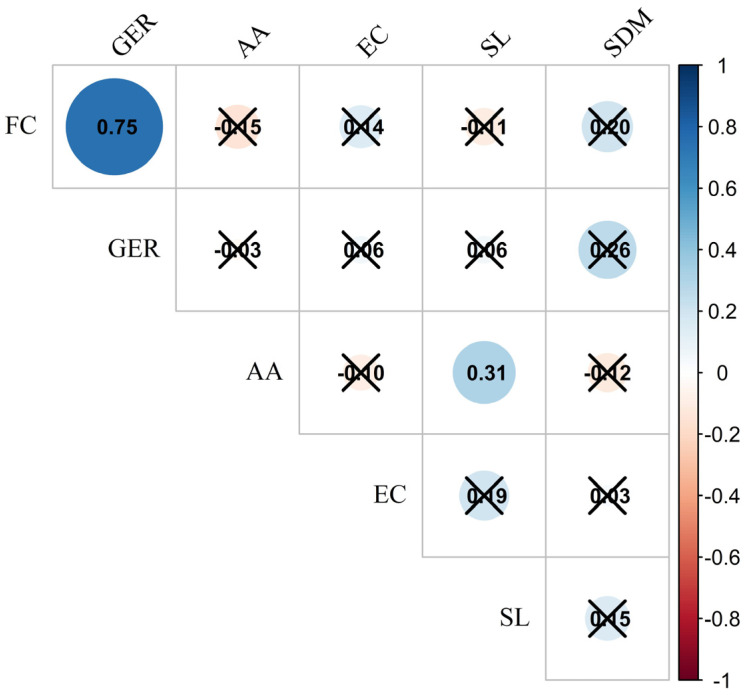
Pearson correlation map among seed physiological quality variables in common bean: first count (FC), germination (GER), accelerated aging (AA), electrical conductivity (EC), seedling length (SL), and seedling dry mass (SDM). non-significant correlation.

Analyzing the Pearson correlation matrix presented in Figure 3, some relevant observations can be made regarding the relationships between the variables evaluated in this study: first count, germination, accelerated aging, electrical conductivity, seedling length, and seedling dry mass.

The strong positive correlation of 0.75 between FC and GER indicates that a higher first count rate (number of seeds germinated in the initial days) is associated with a higher final germination percentage. Conversely, the correlation of 0.31 between AA and EC suggests a moderate positive relationship, indicating that seeds with higher accelerated aging tend to release more electrolytes during imbibition, resulting in higher electrical conductivity. The other variables, such as EC and SL or SDM and the other measures evaluated, did not show significant correlations.

## 4. Discussion

Biological nitrogen fixation is an essential practice for enhancing agricultural sustainability by reducing the need for synthetic fertilizers. Plant response to inoculation and reinoculation may vary depending on the phenological stage, highlighting the need for detailed studies on the influence of microorganism application timing on seed physiological quality.

The results obtained through multivariate analysis of variance indicated significant differences among the treatments evaluated for the first count, germination, and accelerated aging variables. These findings suggest that the method of nitrogen availability to the parent plants directly influences the physiological quality of the produced seeds. As observed by [32], nutrient availability plays a crucial role in embryo and cotyledon formation, directly reflecting on seed vigor and physiological quality.

The results of this study contrast with those of [33], who did not identify significant differences in the physiological quality of seeds from plants subjected to inoculation, pre-planting co-inoculation, and mineral fertilization. This discrepancy highlights the importance of the reinoculation technique, which is emphasized in the present study as a promising strategy to promote seed physiological quality, especially in contexts that favor a more efficient interaction between nitrogen-fixing rhizobacteria and parent plants.

This study is a pioneer in evaluating the physiological quality of seeds from parent plants subjected to inoculation and co-inoculation at different phenological stages. Among the results obtained, the superior performance of the treatment subjected to co-inoculation with *Rhizobium tropici* and *Azospirillum brasilense* at the R5 stage (T7) stands out, presenting averages of 90.50% for the FC and 95% germination potential, surpassing the minimum requirement of 80% established by Normative Instruction No. 45/2013, and showing greater resistance to AA. These results demonstrate the efficiency of fertilization techniques applied at the ideal phenological stage, resulting in seeds with high germination potential.

The results of this study can be applied to other legumes, such as soybean, chickpea, and lentil, which rely on the symbiotic nodulation for nitrogen supply [34]. Co-inoculation in these crops may enhance nodulation, biomass, yield, and seed physiological quality, similar to what was observed in common bean. Therefore, the success of treatment T7 can be attributed to co-inoculation at the R5 stage, a period when the plant begins its reproductive process with shoot formation, characterized by high sensitivity to nutritional and water stress [17]. In this context, the availability of nitrogen-fixing rhizobacteria ensures the maintenance of adequate nutrient levels, meeting the plant’s high demands during this critical developmental phase.

*Azospirillum* stimulates flavonoid secretion, triggering biochemical signals that enhance nodulation [7,35]. Additionally, it solubilizes inorganic phosphorus, increasing its availability to plants and improving crop yield [34]. In co-inoculation, it also promotes the germination and seedling growth of soybean and maize due to the synthesis of plant hormones, benefiting seed quality [18].

Additionally, the contribution of *Azospirillum brasilense* is highlighted as a determining factor in maintaining the adequate nutrition of parent plants, promoting the production of high-quality seeds. This effect is evidenced by the superiority of treatment T7 (co-inoculation with *Rhizobium tropici* + *Azospirillum brasilense* at R5) compared to treatment T3 (reinoculation with *R. tropici* at R5), which did not show the same positive performance in the evaluated variables.

These results corroborate the findings of [32], who, when analyzing the physiological quality of soybean seeds, observed that co-inoculation resulted in seeds with higher germination rates. Thus, the association between nitrogen-fixing and plant growth-promoting rhizobacteria, such as *R. tropici* and *A. brasilense*, stands out as an efficient strategy for improving seed physiological quality, particularly at critical phenological stages such as R5. Furthermore, synergistic interaction between rhizobia and *azospirillum* can enhance biological nitrogen fixation, increasing nitrogen availability for bean crops. This increase in free nitrogen can stimulate the synthesis of essential amino acids and proteins during seed development, positively affecting nitrogen metabolism and, consequently, the quality of the seeds produced.

In the EC test, no statistical significance was observed for the treatments, differing from [36], who evaluated the quality of bean seeds subjected to different nitrogen doses and inoculation. This test establishes that less vigorous seeds, meaning more deteriorated ones, release greater amounts of solutes during imbibition due to a slower restoration of membrane integrity during this period, thus resulting in higher readings [4]. However, it is necessary to highlight that the T10 presented the highest average EC value among the treatments evaluated, confirming the inferior quality of the seed lots produced, despite not differing statistically from the others. These results partly confirm the data obtained in the accelerated aging test.

The electrical conductivity test, also known as electrolyte leakage, is a technique used to assess cold tolerance and tissue damage in plants by measuring the release of electrolytes from cells under stress or injury. This method serves as an indicator of cell membrane stability [37]. As described by [38], greater electrolyte leakage is associated with lower membrane stability. However, in this study, the treatments did not compromise the integrity of the seed cell membranes. Since the test measures solute leaching, the similarity of values among treatments indicates that the physiological quality of the seeds was preserved, regardless of the inoculation technique used.

The absence of significance was also observed for the variables seedling dry mass and seedling length, suggesting a strong trend of genetic influence associated with the intrinsic potential of the evaluated cultivar, as observed by [39]. According to [40], the increase in seedling dry matter mass results from the transfer of reserve tissue compounds to the embryonic axis during germination, and vigorous seeds give rise to plants with greater weight and length. A greater availability of nitrogen may have provided better initial nutrition, as suggested by [41], who indicate that nitrogen directly influences plant growth, biomass formation, and grain yield. Therefore, the research results corroborate previous studies that emphasize the importance of early stages for inoculant application and suggest that reinoculation and co-inoculation may be more effective in phases prior to flowering.

Canonical analysis associates the variables first count and germination, which are tests used to determine seed vigor. Plants originating from high-vigor seeds exhibit superior initial performance compared to those from low-vigor seeds, influencing even productivity [5]. Therefore, the emergence capacity of seeds in the field and the vigor of initial development are of utmost importance to ensure adequate establishment and crop stand, characteristics that are influenced by the plant’s nutritional status [42].

The strong positive correlation between FC and GER indicates that a higher first count rate is associated with a higher final germination percentage. This result is physiologically coherent, as seeds with greater vigor germinate more quickly and uniformly, leading to higher total germination. Studies such as those by [4], reinforce this relationship, associating the first count with seed physiological quality, making it a widely used parameter for assessing initial vigor.

On the other hand, the correlation between AA and EC suggests a moderate positive relationship, indicating that seeds with higher accelerated aging tend to release more electrolytes when imbibed, resulting in higher electrical conductivity. This relationship is explained by the increased permeability of cell membranes in seeds that have suffered greater damage during aging, as described in studies by [31]. This behavior highlights the usefulness of electrical conductivity as an indicator of seed deterioration under stress conditions.

The other variables, such as EC and SL or SDM and the other evaluated measures, did not show significant correlations. This suggests that physiological characteristics related to seed quality, such as electrical conductivity, do not necessarily translate into measurable morphological traits, such as seedling length or biomass, under normal conditions. These results reinforce the importance of combining physiological and physical tests for a comprehensive assessment of seed vigor and quality, considering that each test can capture different aspects of seed and seedling performance.

## 5. Conclusions

The results obtained demonstrate that different inoculation and co-inoculation strategies significantly influence seed physiological quality. Co-inoculation *R. tropici* with *A. brasilense* at the reproductive stage R5 stood out for improving seed germination. The accelerated aging test indicated that co-inoculation at R5 provided greater stress resistance with greater seed vigor, whereas isolated seed inoculation showed higher susceptibility.

The electrical conductivity analysis revealed little variation among treatments, suggesting that seed cell membrane integrity was not significantly affected. Additionally, seedling length and dry mass showed no statistically relevant differences, reinforcing the hypothesis that germination and initial vigor are more critical determinants of final seed quality.

Discriminant and multivariate variance analysis (MANOVA) confirmed statistically significant differences among treatments, highlighting the impact of inoculation strategies on seed physiology. Thus, co-inoculation at reproductive stages emerges as promising approaches to improving seed quality, contributing to more efficient and sustainable agricultural systems.

It is recommended to use co-inoculation with *R. tropici* and *A. brasilense* at the V5 stage, in addition to the inoculation carried out at the time of sowing via seed, in order to obtain batches of bean seeds with superior quality.

## Figures and Tables

**Table 1:** Chemical and physical characteristics of soil collected from the 0–20 cm layer in the experimental area of the Goiás Agency for Technical Assistance, Rural Extension, and Agricultural Research (EMATER), UEG/CCET, Anápolis, Goiás, 2024.

Ca	Mg	Al	H + Al	K	P	Na	Zn	B	Cu	Fe	Mn
-------------cmolc dm^−3^-------------				----mg dm^−3^----		----------------------------mg dm^−3^----------------------------					
2.8	0.7	0	2.9	119.4	12.7	6.4	6.5	0	2.2	26.2	19.3
MO		C	pH	CTC	V	Clay		Silt		Sand	
------------g dm^−3^------------			CaCl_2_	cmolc dm^−3^	%	----------------------------g kg^−1^----------------------------					
28		16.24	5	6.74	56.94	440		110		450	

Ca = calcium, Mg = magnesium, Al = aluminum, H = hydrogen, K = potassium, P (Melich) = phosphorus, Na = sodium, Zn = zinc, B = boron, Cu = copper, Fe = iron, Mn = manganese, V = base saturation, OM = organic matter, C = carbon, pH = hydrogen potential, CEC = cation exchange capacity.

**Table 2:** Description of treatments applied in the 2023/2024 “rainy season” experiment.

Treatments	Description
T1	*Rhizobium tropici* reinoculation at V3 stage
T2	*Rhizobium tropici* reinoculation at V4 stage
T3	*Rhizobium tropici* reinoculation at R5 stage
T4	*Rhizobium tropici* reinoculation at R6 stage
T5	Co-inoculation of *R. tropici* + *A. brasilense* at V3 stage
T6	Co-inoculation of *R. tropici* + *A. brasilense* at V4 stage
T7	Co-inoculation of *R. tropici* + *A. brasilense* at R5 stage
T8	Co-inoculation of *R. tropici* + *A. brasilense* at R6 stage
T9	Control
T10	*R. tropici* seed inoculation
T11	Co-inoculation of *R. tropici* + *A. brasilense* on seeds
T12	Mineral nitrogen fertilization

V3: third fully expanded trifoliate leaf; V4: fourth fully expanded trifoliate leaf; R5: beginning of flowering; R6: formation of the first pods.

**Table 3:** Average values of first count (FC), germination (GER), accelerated aging (AA), electrical conductivity (EC), seedling length (SL), and seedling dry mass (SDM) as a function of the applied treatments.

Treatments	FC (%)	GER (%)	AA (%)	EC (μS cm^−1^ g^−1^)	SL (cm)	SDM (mg seedling^−1^)
T1	74.50 abc	84.00 ab	9.00 fg	115.50 a	19.50 a	0.18 a
T2	63.00 bc	82.50 ab	32.25 abc	118.00 a	21.75 a	0.20 a
T3	72.00 abc	88.50 ab	33.25 abc	113.50 a	18.50 a	0.17 a
T4	28.50 d	76.00 b	22.75 de	104.25 a	20.25 a	0.18 a
T5	57.50 c	77.50 b	29.50 bcd	102.50 a	19.75 a	0.20 a
T6	67.00 abc	88.00 ab	36.50 ab	83.75 a	19.50 a	0.18 a
T7	90.50 a	94.00 a	39.00 a	96.00 a	18.75 a	0.20 a
T8	72.50 abc	85.00 ab	26.25 cd	87.50 a	19.25 a	0.19 a
T9	83.50 ab	94.00 a	38.25 ab	111.00 a	22.25 a	0.20 a
T10	86.00 ab	95.50 a	2.50 g	119.50 a	18.00 a	0.20 a
T11	80.50 abc	87.50 ab	2.25 g	89.00 a	17.75 a	0.20 a
T12	83.50 ab	90.50 ab	13.75 fg	103.50 a	21.75 a	0.21 a

Means followed by the same letter in the column do not differ according to Tukey’s test at a 5% probability level.

## Data Availability

The original contributions presented in this study are included in the article. Further inquiries can be directed to the corresponding author.
